# Preclinical findings: The pharmacological targets and molecular mechanisms of ferulic acid treatment for COVID-19 and osteosarcoma *via* targeting autophagy

**DOI:** 10.3389/fendo.2022.971687

**Published:** 2022-09-20

**Authors:** Guangfu Pang, Tingzhuang Yi, Hongcheng Luo, Lihe Jiang

**Affiliations:** ^1^ School of Basic Medical Science, Youjiang Medical College for Nationalities, Baise, China; ^2^ Department of Oncology, Affiliated Hospital of YouJiang Medical University for Nationalities, Baise, China; ^3^ Department of Medical Laboratory, Affiliated Hospital of Youjiang Medical University for Nationalities, Baise, China; ^4^ School of Basic Medical Sciences, Youjiang Medical University for Nationalities, Baise, China; ^5^ Medical College, Guangxi University, Nanning, China; ^6^ Key Laboratory of Tumor Immunology and Pathology (Army Medical University) Ministry of Education, Chongqing, China

**Keywords:** osteosarcoma, COVID-19, ferulic acid, autophagy, therapeutic targets

## Abstract

The variant virus-based 2019 coronavirus disease (COVID-19) pandemic has reportedly impacted almost all populations globally, characterized by a huge number of infected individuals. Clinical evidence proves that patients with cancer are more easily infected with severe acute respiratory disease coronavirus 2 (SARS-CoV-2) because of immunologic deficiency. Thus, there is an urgent need to develop candidate medications to treat patients with cancer plus COVID-19, including those with osteosarcoma (OS). Ferulic acid, a latent theriacal compound that has anti-tumor and antivirus activities, is discovered to have potential pharmacological use. Thus, in this study, we aimed to screen and determine the potential therapeutic targets of ferulic acid in treating patients with OS plus COVID-19 as well as the pharmacological mechanisms. We applied a well-established integrated methodology, including network pharmacology and molecular docking technique, to detail target prediction, network construction, gene ontology, and pathway enrichment in core targets. The network pharmacology results show that all candidate genes, by targeting autophagy, were the core targets of ferulic acid in treating OS and COVID-19. Through molecular docking analysis, the signal transducer and activator of transcription 3 (STAT3), mitogen-activated protein kinase 1 (MAPK1), and phosphoinositide-3-kinase regulatory subunit 1 (PIK3R1) were identified as the pharmacological targets of ferulic acid in treating OS. These preclinical findings from bioinformatics analysis altogether effectively determined the pharmacological molecules and mechanisms *via* targeting autophagy, demonstrating the therapeutic effectiveness of ferulic acid against COVID-19 and OS.

## Introduction

The 2019 coronavirus disease (COVID-19), reportedly caused by severe acute respiratory disease coronavirus 2 (SARS-CoV-2), has developed into a cosmopolitical pandemic crisis, causing an imponderable death toll (1). The pathogenesis following SARS-CoV-2 infection may comprise acute respiratory distress syndrome and deadly pneumonia, also related to the cytokine storm that induces secondary tissue impairment (2). Despite the availability of COVID-19 vaccines, clinical evidence shows that emerging mutations result in unexpected escape from vaccine effect (3). The protection of existing vaccines may be affected over time against the highly infectious Omicron variant (4). Currently, few specific antiviral medications have been approved for treating COVID-19. From clinical observations, patients with cancer who become infected with SARS-CoV-2 may have a higher risk for in-hospital mortality than other infections (5). In addition, patients with cancer are believed to have a higher risk for severe COVID-19 due to their advanced age and pre-existing disorders (6). Osteosarcoma (OS) is one of the most malignant cancers in orthopedics, occurring in adolescents and the elderly aged >65 years (7). Recent data have reported that the 5-year survival rate of patients with OS has not meliorated in the past 30 years and has approached around 55–75% (8). Children with cancer such as metastatic OS are likely to have a higher risk of developing life-threatening COVID-19 (9). Patients with OS and COVID-19 are medically hard to treat as current clinical treatment is not yet available, and specific functions of the pathogenetic genes involved in OS and COVID-19 are still unreported. In China, traditional Chinese medicine (TCM) is commonly used for chronic clinical disorders, including malignant cancers (10). In anti-COVID-19 practice, the use of TCM for COVID-19 treatment has been recommended in most Chinese provinces, which is characterized by enhanced cure rate (11). Preclinical reports show that some new compounds isolated from TCM have potent antiviral action, including glycyrrhizic acid (12). Ferulic acid, termed 3-methoxy-4-hydroxycinnamic acid, has broad pharmacological properties, such as antioxidant function as well as antibacterial and antiviral actions (13). In addition, the anti-neoplastic effect of ferulic acid has been experimentally validated (14). Furthermore, ferulic acid may mediate potential anti-OS action *in vitro* by promoting the apoptotic pathway (15). Based on the current antiviral and anti-cancer characteristics of ferulic acid, this study was designed to investigate the potential efficacy and mechanisms of ferulic acid in treating OS and COVID-19 by using network pharmacology and molecular docking approaches. The preclinical findings may provide a new direction for the promising treatment of OS and COVID-19 in future clinical practice.

## Materials and methods

### Screening out the OS and COVID-19 targets

Expression profiling by array for OS was retrieved from the Gene Expression Omnibus (GEO, http://www.ncbi.nlm.nih.gov/geo/) database (16), including GSE28424 [modulation of the OS expression phenotype by miRNAs (Illumina)], in which the dataset was based on the platform file GPL13376. Then, the GEO2R online tool (https://www.ncbi.nlm.nih.gov/geo/geo2r/) was applied for gene differential analysis. The screening conditions were set as the difference fold |log_2_FC| >0.5, and the false discovery rate (FDR) was <0.05 to obtain the significantly differentially expressed genes of human OS and to draw the volcano plot associated with differential genes. Using the Genecards database (17) and the Online Mendelian Inheritance in Man (OMIM) database (18), relevant targets of COVID-19 were screened out accordingly.

### Determining ferulic acid-related autophagic targets

The action targets of ferulic acid were obtained using the comparative toxicogenomics database, Swiss Target Prediction database, SuperPred database, and Pharmmapper database. The aforementioned targets were corrected using the reviewed (Swiss-Prot) and Human in the UniprotKB database (19). The OMIM database, GeneCards database, and National Center for Biotechnology Information gene function module were used to identify the autophagy-related targets. Finally, a Venn diagram analysis (20) was performed between the ferulic acid/autophagy-associated targets and OS/COVID-19-related targets to acquire the co-targets of ferulic acid-anti-OS/COVID-19 effect through autophagy.

### GO and KEGG enrichment analyses and visualization

The Gene Ontology (GO) and Kyoto Encyclopedia of Genes and Genomes (KEGG) enrichment analyses and visualization of core targets were performed using R-packages such as “ClusterProfiler”, “org.Hs.eg.Db”, and “ggplot2” in the R language. The gene annotation information was obtained from “org.Hs.eg.Db”, the adjusted *p*-value cutoff was 0.05, and the *q*-value cutoff was 0.05 during enrichment, resulting in an output corresponding to a bubble chart and a circle chart. In addition, Cytoscape_v3.8.2 was used to create a drug-target–GO–CC–MF-pathway disease visualization graph from the results of the biological process and pathway enrichment of ferulic acid against OS and COVID-19 targets through autophagy (21).

### Building up the protein–protein interaction network

To reveal the interaction between the ferulic acid targets and OS/COVID-19 targets, a protein–protein interaction (PPI) network was established through the Search Tool for the Retrieval of Interacting Genes (STRING) database (22). All core targets were transferred to this STRING database. Their size is in direct proportion to the enrichment method. The PPI network was assayed through the network analyzer from Cytoscape v3.7.1 to identify the core genes based on the betweenness, closeness, and degree scores.

### Molecular docking imitation

Molecular docking analysis was performed to illustrate and validate the binding energy of the OS/COVID-19-associated proteins with ferulic acid. The three-dimensional structures of ferulic acid were collected from the PubChem database (https://pubchem.ncbi.nlm.nih.gov/) (23). The crystal structures of the screened proteins, including the signal transducer and activator of transcription 3 (STAT3), mitogen-activated protein kinase 1 (MAPK1), and phosphoinositide-3-kinase regulatory subunit 1 (PIK3R1), were identified from the Uniprot database (https://www.uniprot.org/) and transferred in Protein Data Bank (https://www.pdbus.org/) format. In addition, the Autodock (version 1.5.6) software was used to increase polar hydrogen and set the grid box, and the autodock vina function was further used to conduct the molecular docking assay. The binding energy of ferulic acid with these identified proteins was established, and then the Pymol software was used to visualize the optimal docking conformations (24).

## Results

### Screening for OS and COVID-19 candidate genes

**Figure 1:**
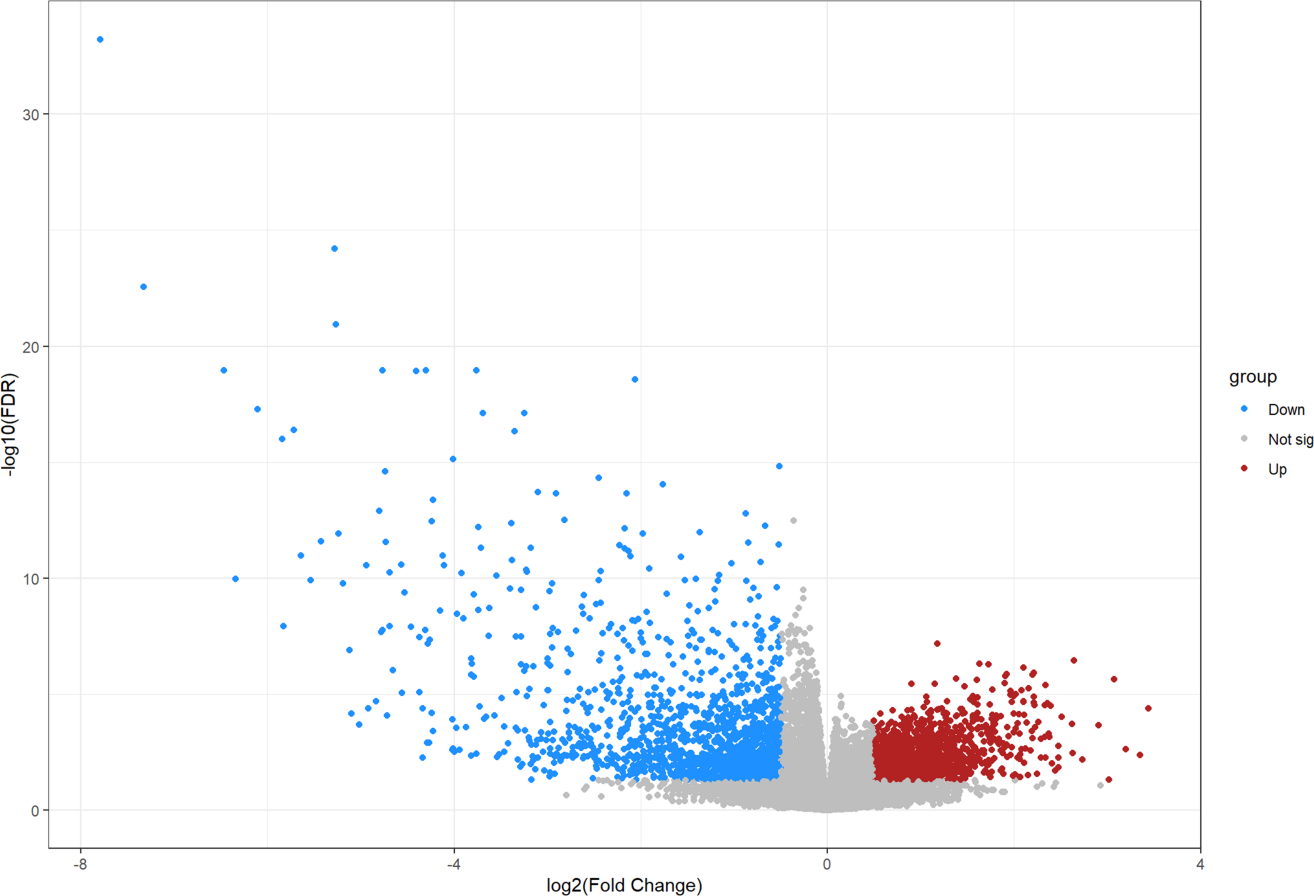
Volcano map illustrating all differentially expressed genes between osteosarcoma and non-osteosarcoma samples.

We compared 19 normal group samples to four OS tumor group samples through the GEO database by retrieving the GSE28424 dataset. After screening, a total of 3,011 differential genes were obtained, including 1,500 upregulated genes and 1,511 downregulated genes, as shown in **Figure 1**. After searching databases such as Genecards, a total of 2,199 relevant COVID-19 target genes were obtained accordingly.

### Ascertaining ferulic acid autophagy-related targets

**Figure 2:**
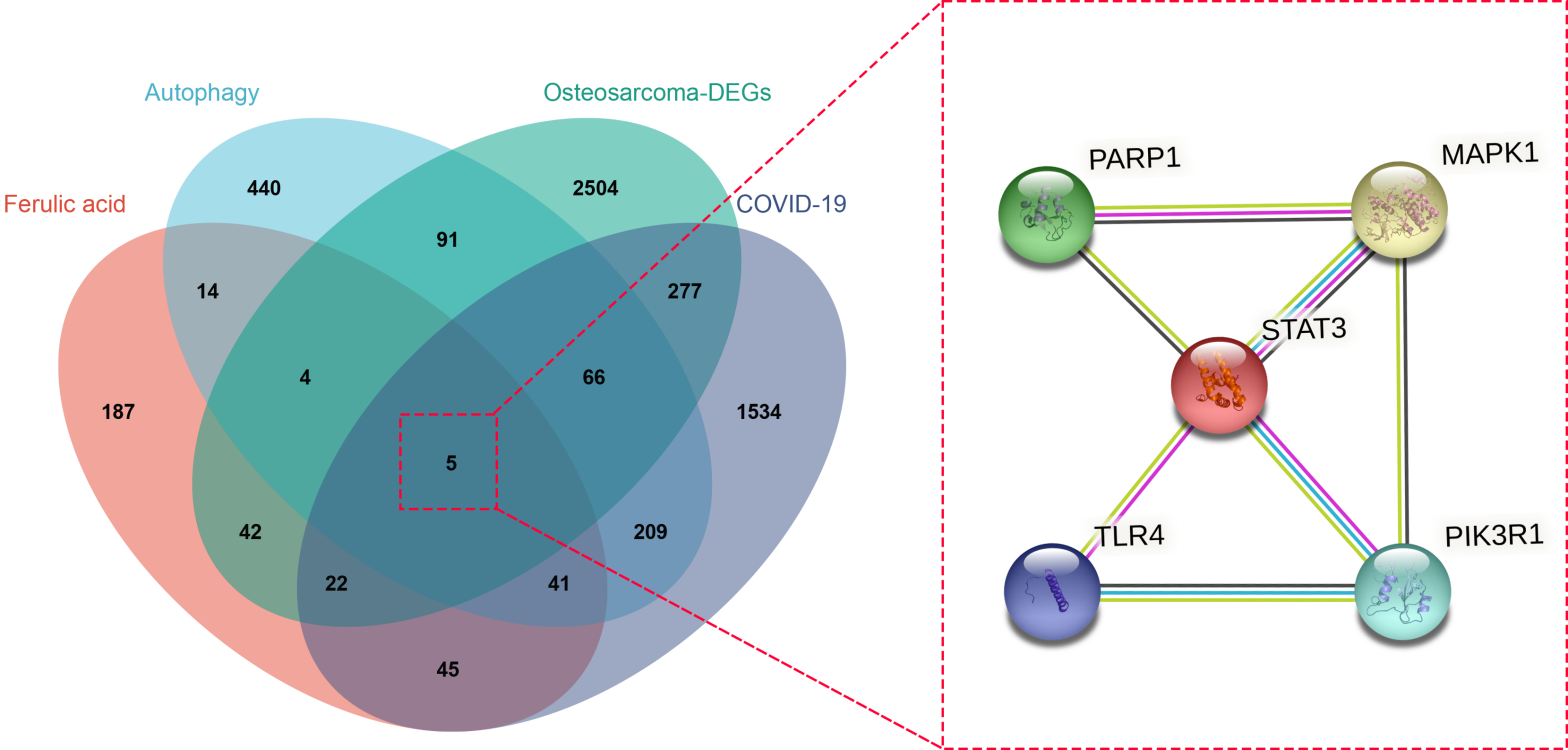
Venn diagram of ferulic acid, autophagy, and osteosarcoma/COVID-19-targeted genes is displayed, and a protein–protein interaction network was established from this diagram.

Using databases such as TCD to search for drug targets and correcting through the UniprotKB database, a total of 360 ferulic acid targets were obtained. The OMIM and other databases were used to obtain 870 autophagy-related targets. The obtained targets were mapped using a Venn diagram, and a total of five intersection targets were obtained, including MAPK1, TLR4, PIK3R1, STAT3, and PARP1 (**Figure 2**).

### Enrichment findings for all intersection targets

**Figure 3:**
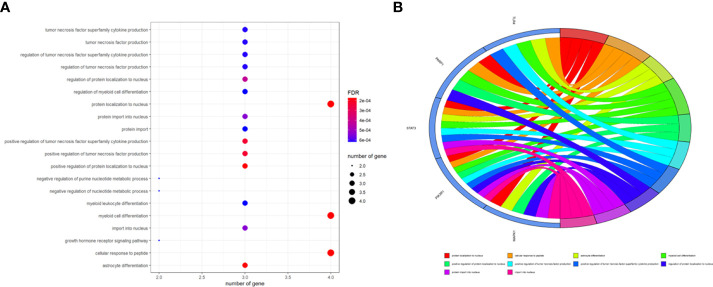
The main terms for biological process, cellular component, and molecular function are presented using bar plots **(A)**. The top terms of the functional enrichment analysis are displayed through the bubble chart **(B)**.

**Figure 4:**
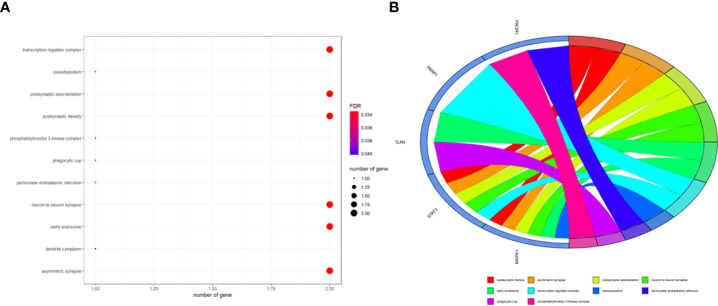
The main terms for biological process, cellular component, and molecular function are presented using bar plots **(A)**. The top terms of the functional enrichment analysis are displayed through the bubble chart **(B)**.

**Figure 5:**
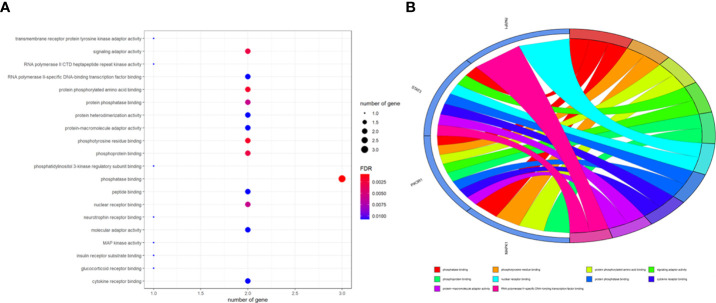
The main terms for biological process, cellular component, and molecular function are presented using bar plots **(A)**. The top terms of the functional enrichment analysis are displayed through the bubble chart **(B)**.

**Figure 6:**
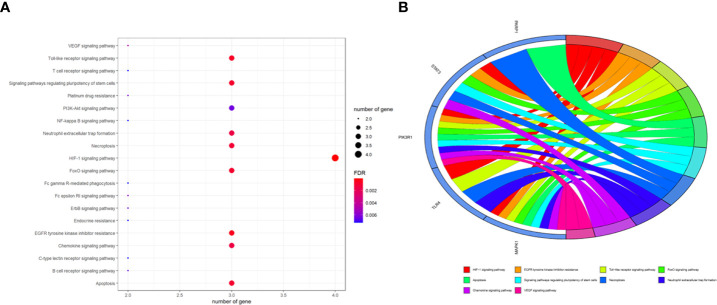
Summary of the Kyoto Encyclopedia of Genes and Genomes enrichment analysis characterized by signaling mechanisms, as shown in bubble chart **(A)** and circle diagram **(B)**.

**Figure 7:**
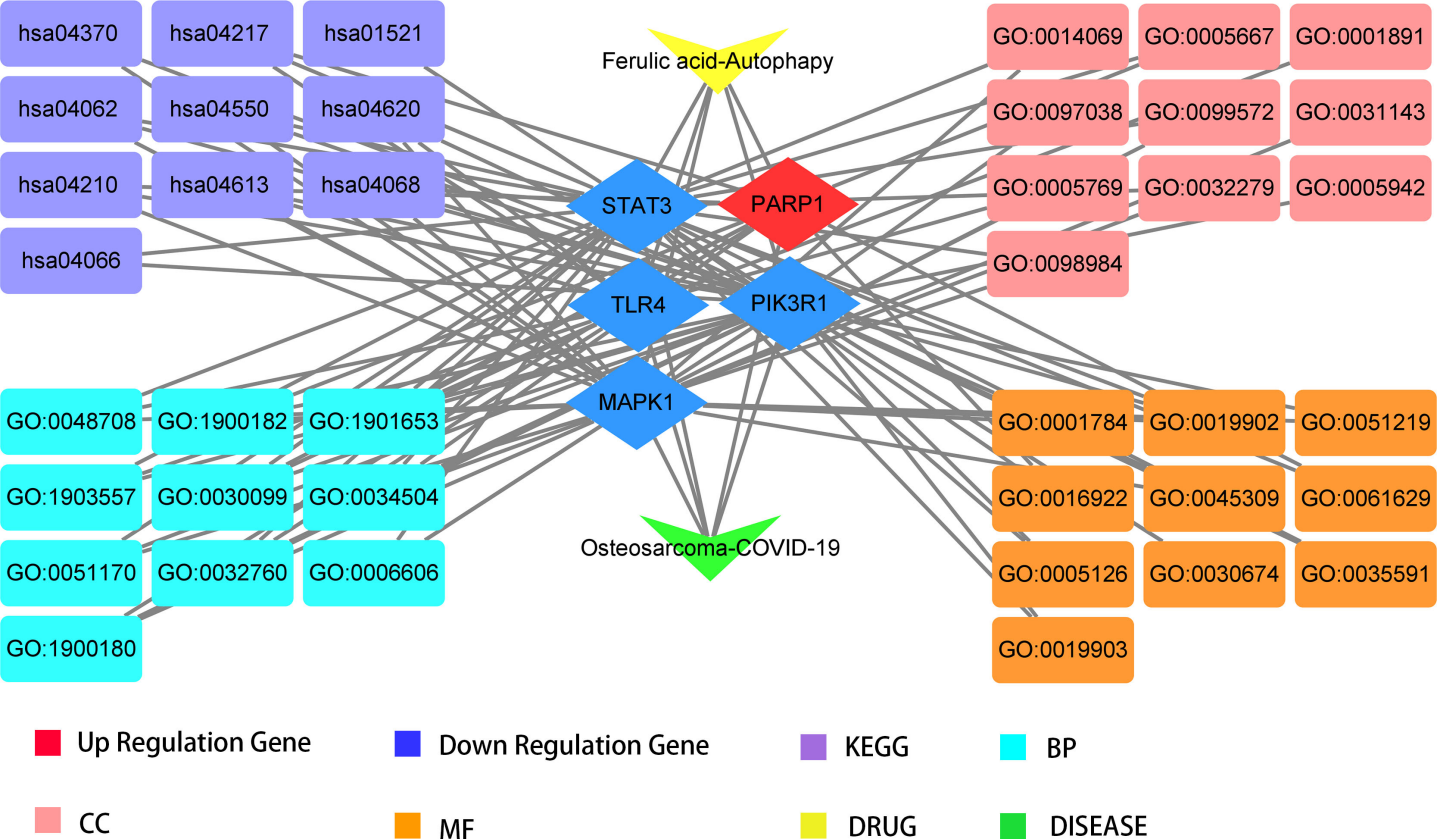
Visualization of the detailed findings in our bioinformatics analysis.

To better indicate the biological characteristics of the identified intersection targets, the R language software was used to implement GO and KEGG enrichment assays. Using parametric analysis, the GO-based biological process (BP) terms (**Figure 3**) mainly comprised protein localization to the nucleus, cellular response to peptide, astrocyte differentiation, myeloid cell differentiation, positive regulation of protein localization to the nucleus, positive regulation of tumor necrosis factor production, positive regulation of tumor necrosis factor superfamily cytokine production, regulation of protein localization to the nucleus, protein import into the nucleus, and import into the nucleus. Furthermore, the key cellular component (CC) terms (**Figure 4**) suggested the functions of postsynaptic density, asymmetric synapse, postsynaptic specialization, neuron to neuron synapse, early endosome, transcription regulator complex, pseudopodium, perinuclear endoplasmic reticulum, phagocytic cup, and phosphatidylinositol 3-kinase complex. Other molecular function (MF) terms (**Figure 5**) indicated phosphatase binding, phosphotyrosine residue binding, protein phosphorylated amino acid binding, signaling adaptor activity, phosphoprotein binding, nuclear receptor binding, protein phosphatase binding, cytokine receptor binding, protein–macromolecule adaptor activity, and RNA polymerase II-specific DNA-binding transcription factor binding. The KEGG enrichment assay was executed to cluster the pharmacological signaling pathways exerted by ferulic acid. A total of 42 molecular pathways with FDR <0.05 were identified (**Figure 6**). These enrichment data indicated the KEGG pathways of ferulic acid, including the HIF-1 signaling pathway, EGFR tyrosine kinase inhibitor resistance, Toll-like receptor signaling pathway, FoxO signaling pathway, apoptosis, signaling pathways regulating the pluripotency of stem cells, necroptosis, neutrophil extracellular trap formation, chemokine signaling pathway, and VEGF signaling pathway. Detailed enrichment terms were visualized in ferulic acid–OS/COVID-19–BP–CC–MF clusters through Cytoscape_v3.8.2 software analysis (**Figure 7**).

### Identification of the core targets

To further ascertain the core targets, topological parameters and target degrees of freedom were measured through Cytoscape_v3.8.2, wherein the filter condition range was set to three to four. As a result, all top three targets, including STAT3, MAPK1, and PIK3R1, were identified as the core genes before further *in silico* imitation analysis.

### Molecular docking data

**Figure 8:**
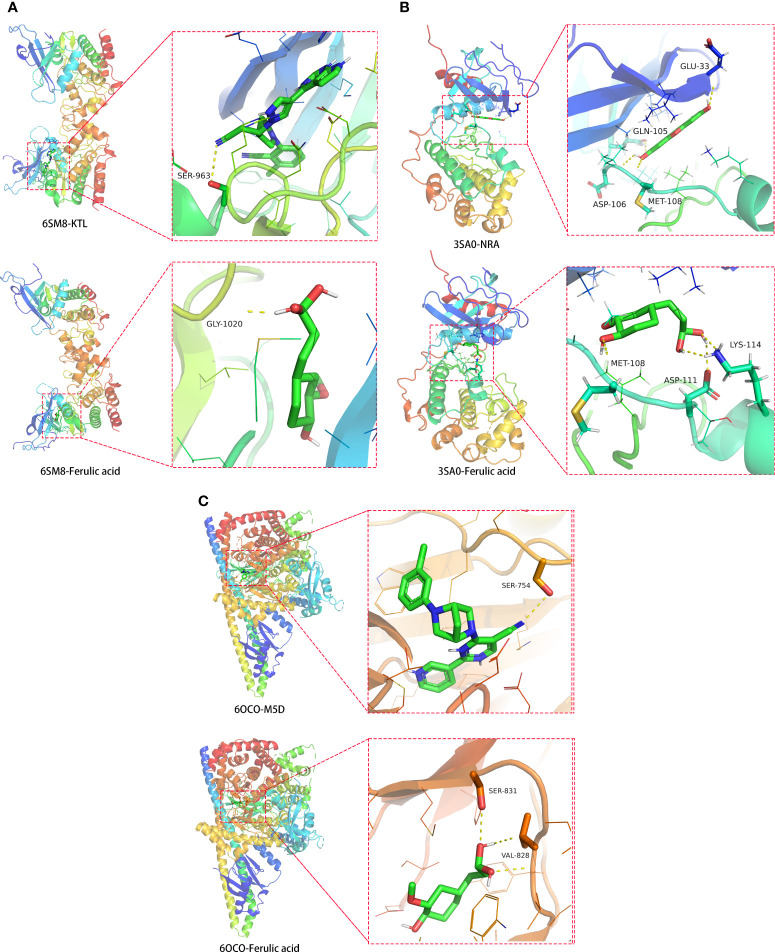
Molecular docking models of ferulic acid binding to the identified STAT3, MAPK1, and PIK3R1 target proteins with respect to **(A)** 6SM8, **(B)** 3SA0, and **(C)** 6OCO.

To confirm the potential targets of ferulic acid, these STAT3, MAPK1, and PIK3R1 proteins were computationally docked with ferulic acid based on the binding energy scores, characterized by 6SM8, 3SA0, and 6OCO binding active sites. The amino acid residue GLY-1020 (3.4 Å) formed a functional hydrogen bond between ferulic acid and 6SM8 (STAT3), and the free docking energy was -6.3 kcal/mol (**Figure 8A**). Moreover, the amino acid residues ASP-111 (2.3 Å), MET-108 (2.3 Å), and LYS-114 (2.4 Å) formed a functional hydrogen bond between ferulic acid and 3SA0 (MAPK1), with a free docking energy of -5.5 kcal/mol (**Figure 8B**). Finally, the amino acid residues SER-831 (3.0 Å) and VAL-828 (2.6 Å) formed a functional hydrogen bond between ferulic acid and 6OCO (PIK3R1), and the free docking energy was -5.7 kcal/mol (**Figure 8C**).

## Discussion

TCM is found to have anti-inflammatory, antiviral, and immunoregulatory activities and is also characterized by a candidate curative effect against COVID-19 (25). It is of interest that bioinformatics analysis through the network pharmacology methodology has demonstrated that niacin may be used to potentially treat colorectal cancer and COVID-19 (21). By using comprehensive bioinformatics approaches, we aimed to reveal the potential molecular mechanisms and therapeutic targets of ferulic acid, a functional molecule, in the treatment of OS and COVID-19. A previous study indicates that ferulic acid has proven antiviral action against PPV infection through effective regulation of the caspase-dependent apoptotic pathway (26). Another *in vitro* experiment suggests that ferulic acid restrains NLRP3 inflammasome activities and lowers the synthesis of inflammatory cytokines through the autophagy channel (27). Thus, ferulic acid may mediate a potential action against COVID-19 by inhibiting virus infection and inflammatory reaction. Furthermore, in the cell culture model of 143B and MG63 OS cells, ferulic acid plays anti-OS effects by inactivating the PI3K/Akt pathway (28). Taking all the present information together, we surmised that ferulic acid may exert a potential action against OS and COVID-19. In our bioinformatics analysis, the clinicopathological features showed 3,011 differentially expressed genes, 1,500 and 1,511 of which were up- and downregulated, respectively. These differentially expressed genes indicate potential diagnostic biomarkers for human OS. In addition, we identified 2,199 genes that are mutual for OS and COVID-19, 360 ferulic acid target genes, and 870 autophagy target genes. Using Venn diagram mapping, we further identified five core ferulic acid targets against OS and COVID-19 *via* autophagy, including MAPK1, TLR4, PIK3R1, STAT3, and PARP1. Among these core target genes, TLR4, STAT3, and PARP1 are associated with the development of COVID-19 (29–31). Moreover, the GO and KEGG enrichment assays revealed that biological processes were distinctly enriched in the positive regulation of tumor necrosis factor production, particularly the positive regulation of tumor necrosis factor superfamily cytokine production. More data have suggested that ferulic acid might inhibit autophagy-dependent cell proliferation in cancers (32) as well as inflammation-related SARS-CoV-2 infection (33). Following the use of the molecular docking analysis, we ultimately identified the core ferulic acid target proteins against OS and COVID-19, namely, STAT3, MAPK1, and PIK3R1. Among these, STAT3 could be developed as a promising therapeutic target for patients with cancer who contract COVID-19 (34). Furthermore, other preclinical evidence shows that both MAPK1 and PIK3R1 could be potential pharmacological targets against human cancers (35, 36). The molecular docking data of these core target proteins suggest that ferulic acid may function in multiple disease stages of patients with cancer plus a SARS-CoV-2 infection. However, our current study still has certain limitations. Experimental validation *in vitro* and *in vivo* was not performed as models of SARS-CoV-2 infection are currently unavailable commercially. Further clinical trials using ferulic acid to treat OS and COVID-19 are still unconducted.

## Conclusions

This study systematically revealed the therapeutic targets and mechanisms of ferulic acid for the potential treatment of OS and COVID-19 through network pharmacology and molecular docking validation. In addition, our results indicate that ferulic acid could regulate multiple anti-cancer and antiviral signaling pathways to exert anti-OS and anti-COVID-19 properties. Our findings conclude that ferulic acid may be a promising therapeutic compound for potentially treating OS and COVID-19. As potential limitation in the current bioinformatics report, we still need further experimental confirmation of our current bioinformatics findings.

## Data availability statement

The original contributions presented in the study are included in the article/**Supplementary Material**. Further inquiries can be directed to the corresponding author.

## Author contributions

LJ conceived and designed the study. GP, TY, and HL performed the data analysis and data interpretation. GP, TY, and HL conducted the bioinformatics and statistical analyses. LJ prepared and revised the manuscript. All authors contributed to the article and approved the submitted version.

## Funding

This research was supported by the Key Laboratory of Tumor Immunology and Pathology Army Medical University (grant no. 2018jsz104) and Guangxi Natural Science Foundation of China (grant no. 2020JJA140172).

## Conflict of interest

The authors declare that the research was conducted in the absence of any commercial or financial relationships that could be construed as a potential conflict of interest.

## Publisher’s note

All claims expressed in this article are solely those of the authors and do not necessarily represent those of their affiliated organizations, or those of the publisher, the editors and the reviewers. Any product that may be evaluated in this article, or claim that may be made by its manufacturer, is not guaranteed or endorsed by the publisher.

## References

[1] Merad M Blish CA Sallusto F Iwasaki A. The immunology and immunopathology of COVID-19. Science. (2022) 375(6585):1122–7. doi: 10.1126/science.abm8108 PMC12828912 35271343

[2] Marc F Moldovan CM Hoza A Magheru S Ciavoi G Farcas DM. Comparative study of cytokine storm treatment in patients with COVID-19 pneumonia using immunomodulators. J Clin Med (2022) 11(10):2945. doi: 10.3390/jcm11102945 35629072 PMC9143723

[3] Kang YF Sun C Sun J Xie C Zhuang Z Xu HQ. Quadrivalent mosaic HexaPro-bearing nanoparticle vaccine protects against infection of SARS-CoV-2 variants. Nat Commun (2022) 13(1):2674. doi: 10.1038/s41467-022-30222-w 35562337 PMC9106700

[4] Garcia-Valtanen P Hope CM Masavuli MG Yeow AEL Balachandran H Mekonnen ZA. SARS-CoV-2 omicron variant escapes neutralizing antibodies and T cell responses more efficiently than other variants in mild COVID-19 convalescents. Cell Rep Med (2022) 3(6):100651. doi: 10.1016/j.xcrm.2022.100651 35654046 PMC9110310

[5] Kodde C Bonsignore M Schöndube D Bauer T Hohenstein S Bollmann A. Mortality in cancer patients with SARS-CoV-2 or seasonal influenza: an observational cohort study from a German-wide hospital network. Infection (2022), 1–9. doi: 10.1007/s15010-022-01852-5 35657531 PMC9163872

[6] Rüthrich MM Giessen-Jung C Borgmann S Classen AY Dolff S Grüner B. COVID-19 in cancer patients: clinical characteristics and outcome-an analysis of the LEOSS registry. Ann Hematol (2021) 100(2):383–93. doi: 10.1007/s00277-020-04328-4 PMC7648543 33159569

[7] Kim HJ Chalmers PN Morris CD. Pediatric osteogenic sarcoma. Curr Opin Pediatr (2010) 22(1):61–6. doi: 10.1097/MOP.0b013e328334581f 19915470

[8] Saraf AJ Fenger JM Roberts RD. Osteosarcoma: Accelerating progress makes for a hopeful future. Front Oncol (2018) 8:4. doi: 10.3389/fonc.2018.00004 29435436 PMC5790793

[9] Offenbacher R Fabish L Baker A Chou AJ Loeb DM. Respiratory failure in a child with pulmonary metastatic osteosarcoma and COVID-19. J Pediatr Hematol Oncol (2021) 43(6):859–60. doi: 10.1097/MPH.0000000000001897 32852398

[10] Oyenihi OR Oyenihi AB Erhabor JO Matsabisa MG Oguntibeju OO. Unravelling the anticancer mechanisms of traditional herbal medicines with metabolomics. Molecules. (2021) 26(21):6541. doi: 10.3390/molecules26216541 34770949 PMC8587539

[11] Zhang DH Zhang X Peng B Deng SQ Wang YF Yang L. Network pharmacology suggests biochemical rationale for treating COVID-19 symptoms with a traditional Chinese medicine. Commun Biol (2020) 3(1):466. doi: 10.1038/s42003-020-01190-y 32811894 PMC7434773

[12] Li R Wu K Li Y Liang X Lai KP Chen J. Integrative pharmacological mechanism of vitamin c combined with glycyrrhizic acid against COVID-19: findings of bioinformatics analyses. Brief Bioinform (2021) 22(2):1161–74. doi: 10.1093/bib/bbaa141 PMC7462346 32662814

[13] Antonopoulou I Sapountzaki E Rova U Christakopoulos P. Ferulic acid from plant biomass: A phytochemical with promising antiviral properties. Front Nutr (2022) 8:777576. doi: 10.3389/fnut.2021.777576 35198583 PMC8860162

[14] Gupta A Singh AK Loka M Pandey AK Bishayee A. Ferulic acid-mediated modulation of apoptotic signaling pathways in cancer. Adv Protein Chem Struct Biol (2021) 125:215–57. doi: 10.1016/bs.apcsb.2020.12.005 33931140

[15] Zhang XD Wu Q Yang SH. Ferulic acid promoting apoptosis in human osteosarcoma cell lines. Pak J Med Sci (2017) 33(1):127–31. doi: 10.12669/pjms.331.12066 PMC5368292 28367185

[16] Barrett T Wilhite SE Ledoux P Evangelista C Kim IF Tomashevsky M. NCBI GEO: Archive for functional genomics data sets–update. Nucleic Acids Res (2013) 41:991–5. doi: 10.1093/nar/gks1193 PMC3531084 23193258

[17] Stelzer G Rosen N Plaschkes I Zimmerman S Twik M Fishilevich S. The GeneCards suite: From gene data mining to disease genome sequence analyses. Curr Protoc Bioinf (2016) 54:1.30.1–1.30.33. doi: 10.1002/cpbi.5 27322403

[18] Hamosh A Scott AF Amberger J Bocchini C Valle D McKusick VA. Online mendelian inheritance in man (OMIM), a knowledgebase of human genes and genetic disorders. Nucleic Acids Res (2002) 30(1):52–5. doi: 10.1093/nar/30.1.52 PMC99152 11752252

[19] UniProt Consortium. UniProt: the universal protein knowledgebase in 2021. Nucleic Acids Res (2021) 49:480–9. doi: 10.1093/nar/gkaa1100 PMC7778908 33237286

[20] Bardou P Mariette J Escudié F Djemiel C Klopp C. Jvenn: an interactive Venn diagram viewer. BMC Bioinf (2014) 15(1):293. doi: 10.1186/1471-2105-15-293 PMC4261873 25176396

[21] Li R Li Y Liang X Yang L Su M Lai KP. Network pharmacology and bioinformatics analyses identify intersection genes of niacin and COVID-19 as potential therapeutic targets. Brief Bioinform (2021) 22:1279–90. doi: 10.1093/bib/bbaa300 PMC7717147 33169132

[22] Szklarczyk D Gable AL Nastou KC Lyon D Kirsch R Pyysalo S. The STRING database in 2021: Customizable protein-protein networks, and functional characterization of user-uploaded gene/measurement sets. Nucleic Acids Res (2021) 49:605–12. doi: 10.1093/nar/gkaa1074 PMC7779004 33237311

[23] Wang Y Bryant SH Cheng T Wang J Gindulyte A Shoemaker BA. PubChem BioAssay: 2017 update. Nucleic Acids Res (2017) 45:955–63. doi: 10.1093/nar/gkw1118 PMC5210581 27899599

[24] Qin X Huang C Wu K Li Y Liang X Su M. Anti-coronavirus disease 2019 (COVID-19) targets and mechanisms of puerarin. J Cell Mol Med (2021) 25:677–85. doi: 10.1111/jcmm.16117 PMC7753316 33241658

[25] Hu Z. COVID-19 patients' views and experiences of traditional Chinese medicine treatment in south Africa. Altern Ther Health Med (2022), AT7248. 35648696

[26] Ma X Guo Z Zhang Z Li X Wang X Liu Y. Ferulic acid isolated from propolis inhibits porcine parvovirus replication potentially through bid-mediate apoptosis. Int Immunopharmacol (2020) 83:106379. doi: 10.1016/j.intimp.2020.106379 32172206

[27] Liu Y Shi L Qiu W Shi Y. Ferulic acid exhibits anti-inflammatory effects by inducing autophagy and blocking NLRP3 inflammasome activation. Mol Cell Toxicol (2022), 1–11. doi: 10.1007/s13273-021-00219-5 PMC8744019 35035494

[28] Wang T Gong X Jiang R Li H Du W Kuang G. Ferulic acid inhibits proliferation and promotes apoptosis *via* blockage of PI3K/Akt pathway in osteosarcoma cell. Am J Transl Res (2016) 8(2):968–80. PMC4846940 27158383

[29] Atalis A Keenum MC Pandey B Beach A Pradhan P Vantucci C. Nanoparticle-delivered TLR4 and RIG-I agonists enhance immune response to SARS-CoV-2 subunit vaccine. J Control Release (2022) 347:476–88. doi: 10.1016/j.jconrel.2022.05.023 PMC9121740 35577151

[30] Luo W Ding R Guo X Zhan T Tang T Fan R. Clinical data mining reveals gancao-banxia as a potential herbal pair against moderate COVID-19 by dual binding to IL-6/STAT3. Comput Biol Med (2022) 145:105457. doi: 10.1016/j.compbiomed.2022.105457 35366469 PMC8957363

[31] Lampropoulou DI Bala VM Zerva E Pliakou E Filippou D Gazouli M. The potential role of the combined PARP-1 and VEGF inhibition in severe SARS-CoV-2 (COVID-19) infection. J Infect Dev Ctries (2022) 16(1):101–11. doi: 10.3855/jidc.15386 35192527

[32] Gao J Yu H Guo W Kong Y Gu L Li Q. The anticancer effects of ferulic acid is associated with induction of cell cycle arrest and autophagy in cervical cancer cells. Cancer Cell Int (2018) 18:102. doi: 10.1186/s12935-018-0595-y 30013454 PMC6045836

[33] DiNicolantonio JJ McCarty MF Assanga SI Lujan LL O'Keefe JH. Ferulic acid and berberine, *via* Sirt1 and AMPK, may act as cell cleansing promoters of healthy longevity. Open Heart (2022) 9(1):e001801. doi: 10.1136/openhrt-2021-001801 35301252 PMC8932268

[34] Bosch-Barrera J Roqué A Teixidor E Carmona-Garcia MC Arbusà A Brunet J. Clinical management of COVID-19 in cancer patients with the STAT3 inhibitor silibinin. Pharm (Basel) (2021) 15(1):19. doi: 10.3390/ph15010019 PMC8778965 35056076

[35] Liu Y Wang D Li Z Li X Jin M Jia N. Pan-cancer analysis on the role of PIK3R1 and PIK3R2 in human tumors. Sci Rep (2022) 12(1):5924. doi: 10.1038/s41598-022-09889-0 35395865 PMC8993854

[36] Yang X Zeng T Liu Z He W Hu M Tang T. Long noncoding RNA GK-IT1 promotes esophageal squamous cell carcinoma by regulating MAPK1 phosphorylation. Cancer Med (2022). doi: 10.1002/cam4.4795 PMC9741976 35608100

